# Association of hyperlipidaemia with 5-year survival after hospitalisation for acute myocardial infarction: a propensity score matched analysis

**DOI:** 10.1136/openhrt-2019-001163

**Published:** 2020-03-17

**Authors:** Mohammed Yousufuddin, Ye Zhu, Ruaa Al Ward, Jessica Peters, Taylor Doyle, Kelsey L Jensen, Zhen Wang, Mohammad Hassan Murad

**Affiliations:** 1 Internal Medicine, Mayo Clinic Health System, Austin, Minnesota, USA; 2 Center for the Science of Health Care Delivery, Mayo Clinic Rochester, Rochester, Minnesota, USA; 3 Health Care Policy & Research, Mayo Clinic, Rochester, Minnesota, USA; 4 Preventive Medicine and Center for the Science of Health Care Delivery, Mayo Clinic Rochester, Rochester, Minnesota, USA

**Keywords:** hyperlipidaemia, mortality, acute myocardial infarction, statin, survival

## Abstract

**Objectives:**

The primary objective was to examine the association between hyperlipidaemia (HLP) and 5-year survival after incident acute myocardial infarction (AMI). The secondary objectives were to assess the effect of HLP on survival to discharge across patient subgroups, and the impact of statin prescription, intensity and long-term statin adherence on 5-year survival.

**Methods:**

Retrospective cohort study of 7071 patients hospitalised for AMI at Mayo Clinic from 2001 through 2011. Of these, 2091 patients with HLP (age (mean±SD) 69.7±13.5) were propensity score matched to 2091 patients without HLP (age 70.6±14.2).

**Results:**

In matched patients, HLP was associated with higher rate of survival to discharge than no HLP (95% vs 91%; log-rank <0.0001). At year 5, the adjusted HR for all-cause mortality in patients with HLP versus no HLP was 0.66 (95% CI 0.58–0.74), and patients with prescription statin versus no statin was 0.24 (95% CI 0.21 to 0.28). The mean survival was 0.35 year greater in patients with HLP than in those with no HLP (95% CI 0.25 to 0.46). Patients with HLP gained on an average 0.17 life year and those treated with statin 0.67 life year at 5 years after AMI. The benefit of concurrent HLP was consistent across study subgroups.

**Conclusions:**

In patients with AMI, concomitant HLP was associated with increased survival and a net gain in life years, independent of survival benefit from statin therapy. The results also reaffirm the role of statin prescription, intensity and adherence in reducing the mortality after incident AMI.

**Key questions**

**What is already known about this subject?**

- Hyperlipidaemia increases the risk of new myocardial infarction among general population. However, the association of hyperlipidaemia with postmyocardial infarction survival in the presence of competing comorbid conditions is not known.

**What does this study add?**

- Among patients with acute myocardial infarction, hyperlipidaemia relative to other key comorbid conditions was associated with increased survival and a net gain in life years independent of survival benefit from statin therapy.

**How might this impact on clinical practice?**

- Among patients with acute myocardial infarction, hyperlipidaemia is a marker of good prognosis relative to other key comorbid conditions.

## Introduction

Hyperlipidaemia (HLP) is a major modifiable risk factor for incident acute myocardial infarction (AMI).^1^ A number of early clinical studies indicated a continuous positive association between cholesterol level and incident AMI,^2^ and lowering of low-density lipoprotein cholesterol (LDL-C) by statin therapy reduced the risk of incident AMI and mortality, often in a dose-dependent fashion.^3^ As a result, more recent guidelines recommended an aggressive reduction in LDL-C using high-intensity statin therapy to further reduce the risk of AMI in select patients.^4^ However, published reports were largely based on observational data and there were no randomised controlled trials that have evaluated the effect of specific LDL-C target or head-to-head comparison of multiple LDL-C targets on clinical outcomes.^4^ Results from recent large observational studies support an inverse association wherein a diagnosis of HLP, counterintuitively, conferred a survival advantage after AMI.^5–7^ Patients with incident AMI show a wide interindividual variability in their subsequent survival, potentially attributable to differences in the prevalence of comorbid conditions (CCs).^8^ The ability to accurately predict survival in an individual patient at the time of hospitalisation is essential to provide a personalised care plan.

To date, the studies focused on the influence of baseline HLP on long-term survival after AMI are lacking. On the contrary, a few observational studies suggested that baseline HLP was associated with reduced all-cause mortality after AMI, but these analyses did not account for numerous CCs which are generally prevalent in patients with AMI.^6 7 9^ We, therefore, sought to examine the association between baseline HLP and 5-year survival in a cohort of patients who were hospitalised for incident AMI with additional focus on the relative importance of other prognostic indicators, such as age, gender, race, comorbidity and statin use on 5-year survival rates. We also examined the effect of adherence to statin on postmyocardial infarction mortality and 5-year survival. To minimise the differences in baseline characteristics, we assembled balanced pairs of patients with or without HLP using propensity scores.

## Methods

### Study population and data collection

The study cohorts comprised of adults aged ≥18 years, admitted to Mayo Clinic, Rochester, Minnesota with a discharge diagnoses of AMI, either ST-elevation myocardial infarction (STEMI) or non-STEMI (NSTEMI), with first hospitalisation during the study period included in the analysis. Patients in whom primary discharge diagnosis was not AMI were not included in the study. Discharge diagnoses were identified by the *International Classification of Diseases, Ninth Revision, Clinical Modification* (ICD-9-CM) codes (online supplementary table 1). We limited the study enrolment period from 1 August 2001 to 31 July 2011 to allow prespecified minimum of 5-year follow-up. Patients with index hospitalisation for AMI during the study were recruited regardless of pre-existing coronary artery disease (CAD) or prior AMI. For patients with multiple hospitalisations for AMI beyond the index event, the first event was incorporated in analysis. Patients who refused participation in clinical trials and those outside the Mayo Clinic catchment area were excluded. Further details of data extraction are published elsewhere.^10^ The study was approved by the Mayo Clinic Institutional Review Board and need for patient consent was waived.

10.1136/openhrt-2019-001163.supp1 Supplementary data

### Measurement of outcomes

The primary outcome was 5-year survival after index hospitalisation for AMI. The secondary outcomes were (1) survival to discharge, (2) 5-year mortality across study subgroups (age <65 years vs ≥65 years, male vs female, white vs non-white, normal vs low left ventricular ejection fraction (LVEF), revascularisation vs no revascularisation with percutaneous coronary intervention (PCI), revascularisation vs no revascularisation with coronary artery bypass surgery (CABG)) and (3) impact of statin prescription, intensity and long-term statin adherence on survival to 5 years after AMI. We also examined the association between total cholesterol (TC), high-density lipoprotein cholesterol (HDL-C), non-HDL-C or triglyceride, as binary variables in accordance with professional societies’ guidelines,^11 12^ and 5-year mortality after index hospitalisation for AMI.

### Ascertainment of AMI

For each patient, AMI (STEMI or NSTEMI) as the primary diagnosis at the time of discharge was documented by the attending physician and then captured by data abstractors.

### Ascertainment of HLP

Contemporary guidelines recommend assessment of total cardiovascular risk for secondary prevention of cardiovascular risk and initiation of statin in patients who had AMI, given insufficient evidence for specific LDL-C, non-HDL-C, HDL-C, TC or triglyceride thresholds.^11 12^ Patients with AMI are generally considered as very high-risk for subsequent cardiovascular events. Current guidelines recommend intensifying lipid lowering therapy with non-statin drugs if statin alone fail to achieve a LDL-C level <70 mg/dL. In the current study, HLP was defined as provider documented and identified as secondary diagnosis using ICD-9-CM codes as represented in online supplementary table 1, or a new in-hospital diagnosis based on LDL-C level ≥100 mg/dL during index hospitalisation or within the preceding 6 months. The physician-reported diagnosis of HLP at baseline was based on then clinical practice in accordance with National Cholesterol Education Programme Expert Panel on Detection, Evaluation and Treatment of High Blood Cholesterol in Adults (Adult Treatment Panel III).^13^ For most primary care physicians HLP was a TC ≥240 mg/dL, a LDL-C ≥100 mg/dL or a HDL- C <40 mg/dL with or without hypertriglyceridaemia (triglycerides >200 mg/dL).^14^

LDL-C was measured indirectly by the Friedewald method.^15^ Published reports suggested that lipid panels measured within the first 24 hours after an acute cardiovascular event reliably represents baseline level.^16^

### Ascertainment of CCs

We considered a CC to be present if it was documented as a secondary diagnosis during index hospitalisation. We determined a panel of 20 CCs^17^ by Clinical Classifications Software (CCS) codes developed by US Healthcare Cost and Utilization Project. CCs with low prevalence (<3%) were excluded from the data analysis. Several observational studies demonstrated lower prevalence of certain CCs at least partly attributable to coding practices, physician and patient-reported bias and acute conditions prioritisation bias.^18–21^ Observational studies demonstrated that certain CCs were underreported while others were accurately reported.^19 22^

### Ascertainment of mortality

All deaths occurring from admission to censoring date were abstracted from Mayo Clinic electronic medical records.

### Ascertainment of statin and non-statin HLP therapy

Statin and non-statin drug (ezetimibe, fibrates or niacin) prescription at discharge including statin prescription by intensity was captured from electronic medical records. Intensity of a statin therapy was defined as high-intensity (atorvastatin 40 mg to 80 mg and rosuvastatin 20 mg to 40 mg) and non-high intensity.^23^

### Ascertainment of adherence to statin

Adherence to statin was defined as medication possession ratio (MPR) between the first and last prescription^24^ as documented in the medical records. MPR for statin was calculated as number of days statin received after hospitalisation for AMI divided by number of days followed until death or a 5-year follow-up period. Adherence was stratified into three levels according to MPR: low (<50%), intermediate (50%–79%) and high adherent (≥80% or greater) groups.

### Statistical analysis

Student’s t-test, Wilcoxon rank-sum test and χ^2^ test were used to compare means, medians and proportions, respectively.

#### Propensity score analysis

Propensity scores were estimated using logistic regression (PROC PS MATCH in SAS).

#### Covariate selection in propensity score matching

We chose age, gender, length of hospital stay, race, select CCs (cancer, chronic kidney disease (CKD), chronic obstructive pulmonary disease (COPD), diabetes mellitus, hypertension and stroke) and year of hospitalisation as covariates for propensity score matching based on prior knowledge of their respective association with clinical outcome following AMI. Prior studies showed that propensity score modelling based on covariates that impact clinical outcome results in accurate effect estimates.^25^

#### Multivariable Cox models

Cox proportional hazards models were performed on the matched samples using a robust variance estimator to account for matching.

#### Survival analysis

Cox proportional hazard model fitted survival curves stratified by HLP were generated for the entire cohort and STEMI and NSTEMI subgroups. Restricted mean survival time (RMST) method was used to estimate survival time for each patient.^26^ Differences in survival time between patients with and without HLP were compared using t-test. When calculating the life years lost or gained at 5-year time interval for patient subgroups, group means were used as a baseline survival time. Separate Cox regression model fitted survival curves were constructed to elucidate the relationship between level of statin adherence and time to death. Similarly, we estimated survival time according to levels of statin adherence by separate RMST analysis. Data analyses were performed using SAS 9.4 version (V.9.4, Cary, North Carolina, USA) and Stata (MP15.1, College Station, Texas, USA).

#### Sensitivity analysis

Analysis restricted to patients with no statin prescription at discharge was performed.

## Results

### Study population and baseline characteristics

Online supplementary figure 1 illustrates the Strengthening the Reporting of Observational Studies in Epidemiology flow diagram for selection of final study cohorts. Initial prematched study cohort comprised of 7071 patients with AMI, 4809 (68%) had concurrent HLP and 2262 (32%) had no HLP. Using propensity scores, 2091 patients with HLP (age (mean±SD) 69.7±13.5, male 63%, white 90%) were matched to 2091 without HLP (age 70.6±14.2, male 63%, white 90%). After propensity score matching, the two groups were similar in baseline characteristics with absolute standardised difference between 0.006 and 0.11 and an exact match on gender and race, thereby suggesting that the variables were well balanced between patients with and those without HLP (table 1). Matched patients with incomplete data were excluded from regression analysis. Of 20 CCs, only 7 CCs were included in final analysis for their frequency ≥3%.

**Table 1:** Patient characteristics and standardised differences before and after propensity score matching

	All patients n=7071			Propensity score matched patients n=3546		
	HLP n=4809	No HLP n=2262	Absolute standardised difference	AMI with HLP n= 2091	AMI with no HLP n=	Absolute Standardised Difference
Demographics						
Age, years (mean±SD)	67.1±13.8	71.1±14.0	0.287	69.7±13.5	70.6±14.2	0.066
Male, n (%)	3255 (68)	1419 (63)	0.104	1325 (63)	1325 (63)	0
White, n (%)	4458 (93)	2011 (89)	0.132	1887 (90)	1887 (90)	0
Anthropometric measurements						
BMI (kg/m^2^)	30.0±6.2	28.9±6.6		29.4±6.2	29.0±6.7	
BMI, missing n (%)	743 (16)	540 (24)		532 (25)	472 (23)	
Clinical characteristics						
LOS, days, median (quartiles 25%–75%)	3.0 (2 to 5)	4.0 (3 to 8)	0.294	4.0 (3 to 6)	4.0 (3 to 7)	0.107
Year of hospital admission						
2001, n (%)	146 (3)	190 (8)	0.752	141 (7)	145 (7)	
2002, n (%)	355 (7)	375 (17)		336 (16)	327 (16)	
2003, n (%)	325 (7)	435 (19)		287 (14)	395 (19)	
2004, n (%)	474 (10)	368 (16)		323 (15)	351 (17)	
2005, n = (%)	581 (12)	245 (11)		330 (16)	232 (11)	
2006, n (%)	555 (12)	179 (8)		221 (11)	173 (8)	
2007, n (%)	537 (11)	131 (6)		160 (8)	131 (6)	
2008, n (%)	492 (10)	122 (5)		101 (5)	120 (6)	
2009, n (%)	462 (10)	96 (4)		83 (4)	96 (5)	
2010, n (%)	548 (11)	80 (4)		75 (4)	80 (4)	
2011 n = (%)	334 (7)	41 (2)		34 (2)	41 (2)	0.029
Comorbid conditions						
Cancer, n (%)	378 (8)	187 (8)	0.015	187 (9)	173 (8)	0.025
CKD, n (%)	470 (10)	221 (10)	0.0001	194 (9)	198 (10)	0.006
COPD, n (%)	430 (9)	302 (13)	0.14	256 (12)	265 (13)	0.014
Diabetes, n (%)	1388 (29)	598 (26)	0.054	525 (25)	551 (26)	0.028
Heart failure, n (%)	832 (17)	601 (27)	0.225	482 (23)	530 (25)	0.056
Hypertension, n (%)	3376 (70)	1296 (57)	0.271	1264 (60)	1240 (59)	0.02409
Stroke, n (%)	192 (4)	74 (3)	0.039	69 (3)	73 (4)	−0.01023
Lipid levels						
LDL-C (mg/dL)	112.8±38.4	73.2±18.7		117.9±37.4	73.5±18.7	
LDL-C, missing data n (%)	240 (5)	467 (21)		101 (5)	424 (20)	
Drug treatment						
Statin, n (%)	2714 (56)	795 (35)	0.437	814 (39)	783 (37)	0.03046

BMI, body mass index; CKD, chronic kidney disease; COPD, chronic obstructive pulmonary disease; LDL-C, low-density lipoprotein cholesterol; LOS, length of stay.

### Survival

#### Effect of HLP on survival

**Figure 1:**
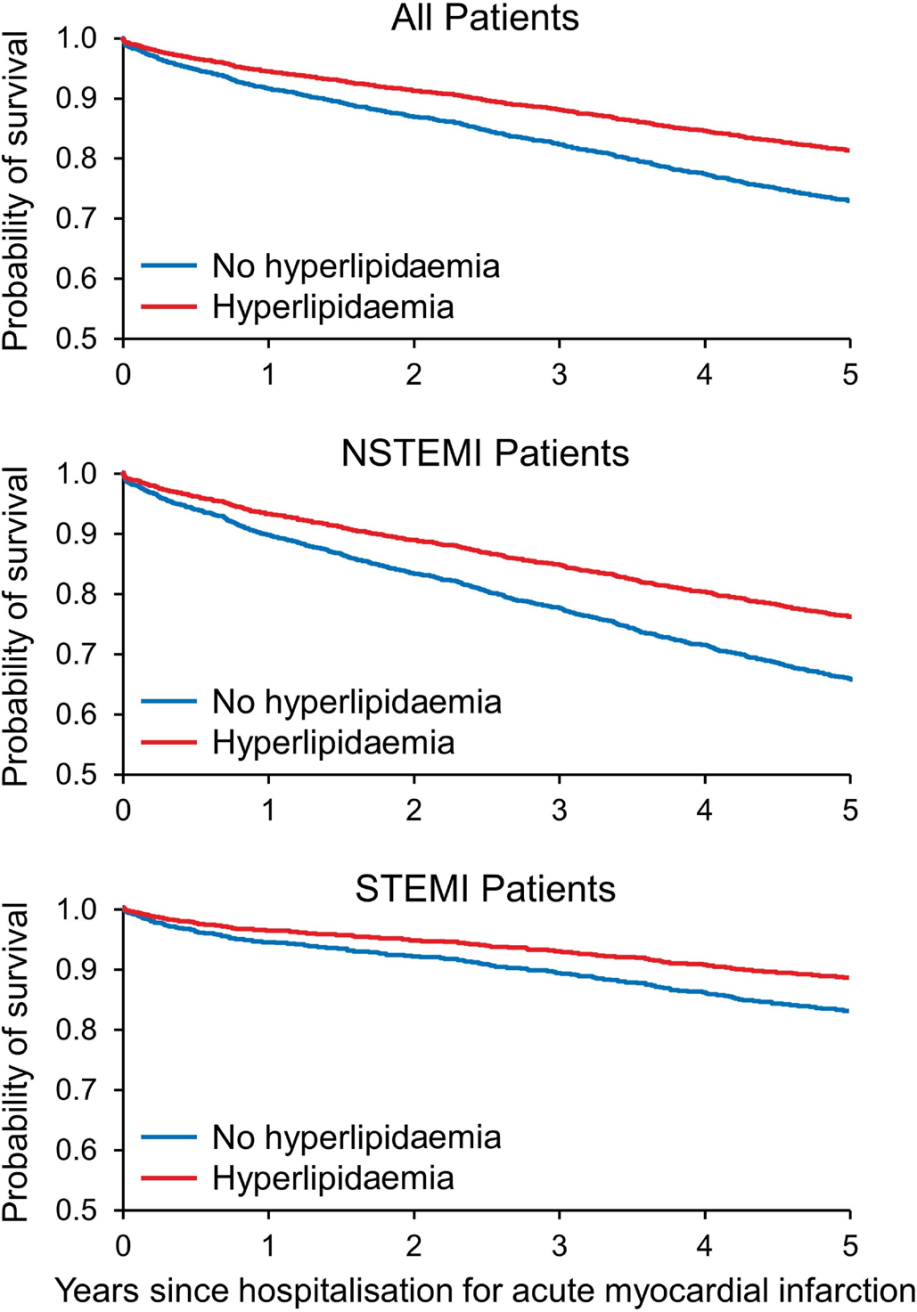
Survival curves by Cox regression model for patients with hyperlipidaemia and those matched with no hyperlipidaemia at 5 years after acute myocardial infarction.

The survival benefit of having HLP is represented by Cox proportional hazard model fitted survival curves (figure 1). Patients with HLP had higher rate of survival to hospital discharge than those with no HLP (95% vs 91%; log-rank p<0.0001; online supplementary figure 2). The differences in survival to discharge between patients with HLP and those with no HLP was consistent across STEMI (95% vs 89%, log-rank p<0.0001) and NSTEMI (95% vs 92%, log-rank p<0.0003) subgroups. The cumulative 5-year survival was 70% (2499 of 3546) in overall patients, 65% (1399 of 2155) in NSTEMI and 79% (1100 of 1391) in STEMI. The absolute estimated difference in length of survival over 5-year follow-up in patients with versus without HLP was 0.35 year (95% CI 0.25–0.46) for overall patients, 0.39 year (95% CI 0.26 to 0.53) for NSTEMI and 0.24 year (95% CI 0.09 to 0.39) for STEMI. In separate analysis patients with HLP gained on an average 0.17 life year (95% CI 0.11 to 0.24) in overall patients, 0.20 life year (95% CI 0.11 to 0.29) in NSTEMI and 0.11 life year (95% CI 0.02 to 0.20) in STEMI over a 5-year follow-up. These survival benefits conferred by HLP in patients with AMI were independent of statin use, demographics and a number of CCs. Table 2Table 2 summarises the results of restriction mean survival time.

**Table 2:** Results of restriction mean survival time: estimates of differences in length of survival and life years gained over 5 years after incident AMI in patients with HLP vs no HLP

Study cohorts	Difference in length of survival in years, HLP vs no HLP Mean (95% CI)	Gain in life in years with HLP Mean (95% CI)	Gain in life in years with statin Mean (95% CI)
Entire cohort	0.35 (0.25 to 0.46)	0.17 (0.11 to 0.24)	0.67 (0.62 to 0.71)
NSTEMI	0.39 (0.26 to 0.53)	0.20 (0.11 to 0.29)	0.77 (0.70 to 0.83)
STEMI	0.24 (0.09 to 0.39)	0.11 (0.02 to 0.20)	0.48 (0.42 to 0.54)
No statin group	0.53 (0.38 to 0.68)	0.26 (0.16 to 0.36)	

Patients with AMI with concomitant HLP had a greater mean survival and a gain in life years over 5-year time compared with those with no HLP.

AMI, acute myocardial infarction; HLP, hyperlipidaemia; NSTEMI, non-ST-elevation myocardial infarction; STEMI, ST-elevation myocardial infarction.

#### Effect of prescription statin at discharge on survival

Prescription statin at discharge added 0.67 life year (95% CI 0.62 to 0.71) in overall cohort, 0.77 life year (95% CI 0.70 to 0.83) in NSTEMI and 0.48 life year (95% CI 0.42 to 0.54) in STEMI.

#### Statin intensity

Of 2875 patients who received statin therapy on discharge, 289 (10%) were on high-intensity statin. Multivariable Cox regression analysis showed that high-intensity statin was independently associated with lower 5-year mortality compared with non-high-intensity statin therapy (HR 0.52, 95% CI 0.42 to 0.63, p<0.0001)

#### Non-statin lipid lowering therapy

Non-statin lipid lowering medications including ezetimibe (n=16, 0.4%), niacin (n=60, 1.4%) and fibrates (n=56, 1.3%) were prescribed in small numbers of study population. The numbers were too small to impact the outcome.

#### Effect of statin adherence during follow-up on survival

**Figure 2:**
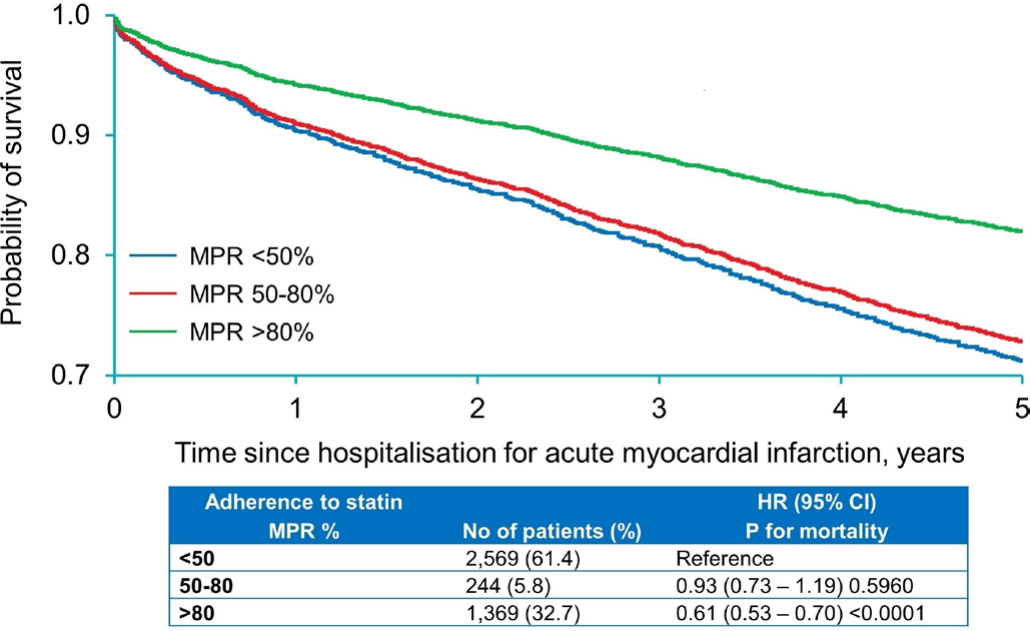
Survival curves by Cox regression model for patients with acute myocardial infarction by statin adherence subgroups defined as low, intermediate and high adherence groups according to MPR. Adjusted HR, 95% CI and p values for 5-year mortality among patients hospitalised for acute myocardial infarction by statin adherence subgroups. MPR, medication possession ratio.

In patients who received statin prescription at discharge, 61.4%, 5.8% and 32.7% patients had low (<50%), intermediate (50%–79%) and high (≥80%) MPRs, respectively. In Cox adjusted model, the high and intermediate adherent groups were compared with the referent low adherent group. Compared with low statin adherence, high adherence was independently associated with a 39% lower risk of death (HR 0.61, 95% CI 0.53 to 0.70, p<0.0001; figure 2). No difference in mortality was found between patients with intermediate and low levels of statin adherence. High level of statin adherence was associated with an absolute 0.35 year survival benefit compared with low level of statin adherence (figure 2).

#### Sensitivity analysis

Sensitivity analysis excluding patients who were prescribed statin at discharge, showed a persistence of survival benefit with HLP compared with no HLP with absolute difference on length of survival of 0.53 year (95% CI 0.38 to 0.68) and 0.26 life year (95% CI 0.16 to 0.36) gain during the 5 years of follow-up period.

### Mortality

**Figure 3:**
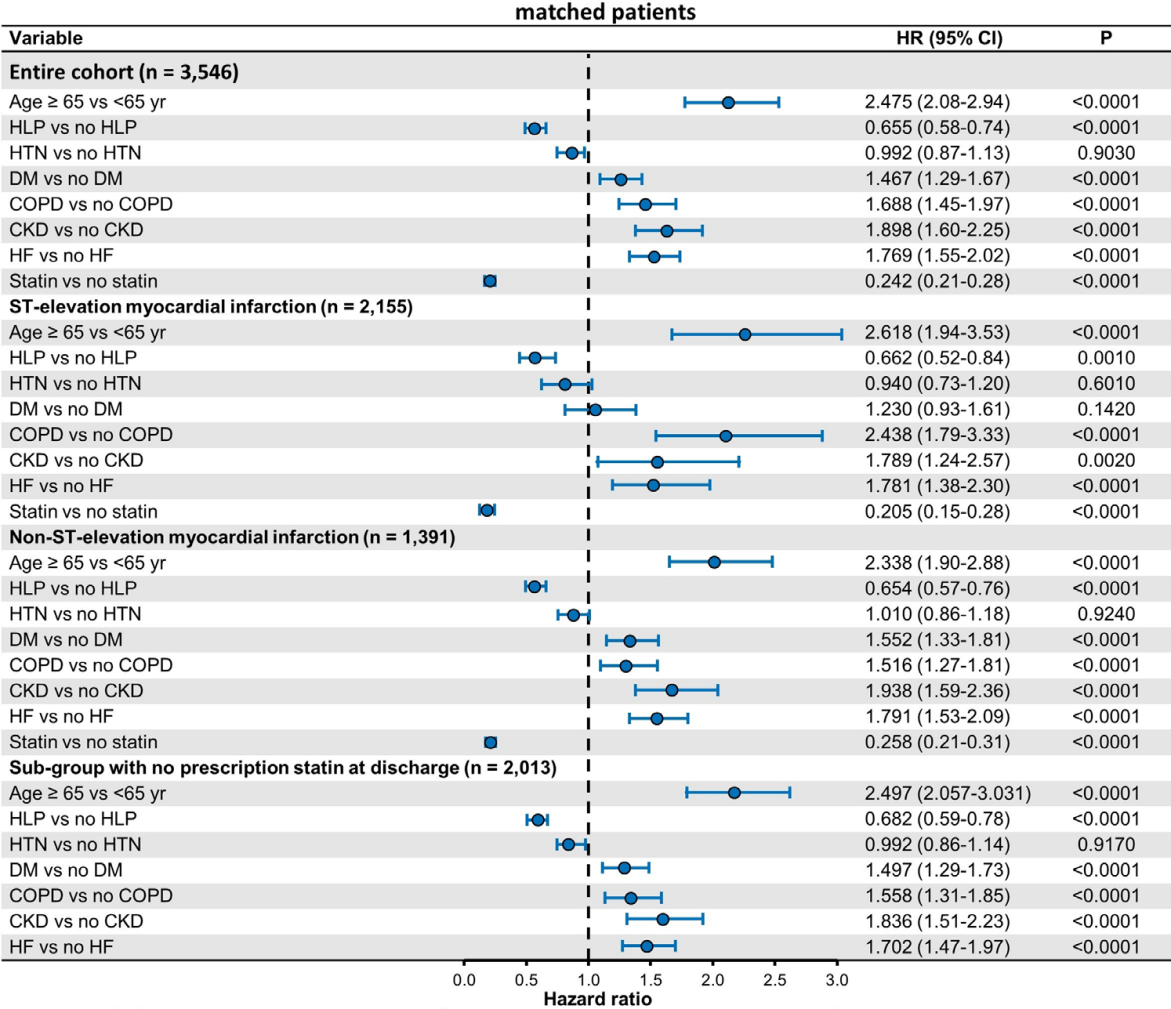
Results of Cox regression analysis, adjusted HR for all-cause mortality among propensity score matched patients. CKD, chronic kidney disease; COPD, chronic obstructive pulmonary disease; DM, diabetes mellitus; HF, heart failure; HLP, hyperlipidaemia; HTN, hypertension.

There were 1050 (29%) all-cause deaths in entire cohort including 759 (35%) in NSTEMI and 291 (21%) in STEMI at 5 years. The adjusted HR for all-cause mortality in patients with HLP vs no HLP was 0.66 (95% CI 0.58 to 0.74) in overall patients, 0.65 (95% CI 0.56 to 0.76) in NSTEMI and 0.66 (0.52–0.84) in STEMI. Prescription statin compared with no statin at discharge was associated with lower mortality rates (HR 0.24, 95% CI 0.21 to 0.28 for overall patients; HR 0.26, 95% CI 0.21 to 0.31 for NSTEMI; HR 0.20, 95% CI 0.15 to 0.28 for STEMI). Figure 3 represents results of Cox regression analysis. Patients with NSTEMI were older than those with STEMI and had significantly higher prevalence of cancer, COPD, diabetes mellitus, CKD, stroke and heart failure likely contributing to a higher mortality in NSTEMI than in STEMI.

### Subgroup analysis

Multivariable-adjusted Cox regression model showed HLP to be independently associated with lower in-hospital mortality after AMI compared with no HLP (HR 0.58, 95% CI 0.45 to 0.74, p<0.0001). These results are presented in online supplementary figure 2. The association between HLP and 5-year mortality remained consistent across age groups (<65 years, HR 0.60, 95% CI 0.49 to 0.73; ≥65 years, HR 0.72, 95% CI 0.66 to 0.79), both sexes (male, HR 0.68, 95% CI 0.61 to 0.75; female, HR 0.76, 95% CI 0.67 to 0.87), racial groups (white, HR 0.70, 95% CI 0.64 to 0.77; non-white, HR 0.75, 95% CI 0.57 to 0.98), low versus normal LVEF (LVEF≥50, HR 0.69, 95% CI 0.60 to 0.79; LVEF≤49, HR 0.69, 95% CI 0.61 to 0.78), statin versus no statin on dismissal (statin, HR 0.66, 95% CI 0.59 to 0.74; no statin, HR 0.81, 95% CI 0.72 to 0.92), PCI versus no PCI (PCI, HR 0.73, 95% CI 0.64 to 0.82; no PCI, HR 0.72, 95% CI 0.65 to 0.81) and in no CABG (HR 0.69, 95% CI 0.63 to 0.76). However, in CABG group, patients with HLP showed lower 5-year mortality trend compared with those with no HLP (HR 0.84, 95% CI 0.68 to 1.04). The results are presented in online supplementary table 3.

Patients who had other lipid fractions measured were stratified into prespecified subgroups according to their respective concentration. We performed separate multivariable Cox regression models to assess their independent effect on 5-year mortality. We found no independent associations of TC (TC ≤180 mg/dL vs ≥181 mg/dL, HR 0.90, 95% CI 0.81 to 1.01, p=0.0647), HDL-C (HDL-C ≥45 mg/dL vs ≤44 mg/dL, HR 0.97, 95% CI 0.88 to 1.06, p=0.5251) and triglycerides (triglyceride ≤200 mg/dL vs ≥201 mg/dL, HR 1.04, 95% CI 0.93 to 1.17, p=0.4813) with 5-year mortality after AMI. However, elevated non-HDL-C was associated with lower 5-year mortality after AMI (non-HDL-C ≥130 mg/dL vs ≤129 mg/dL, HR 0.84, 95% CI 0.76 to 0.92, p=0.0002).

## Discussion

### Main findings

Our main findings in patients hospitalised for AMI were as follows: concomitant HLP, compared with no HLP was associated with higher rates of survival to hospital discharge and survival to 5 years controlling for patient-level characteristics. We also obtained precise estimates of the association between HLP and survival to determine the absolute difference in 5-year survival after AMI in patients with HLP versus no HLP. The analysis showed an absolute increase in survival and a gain in life years over 5 years among patients with HLP versus no HLP after AMI. These survival benefits of having HLP at baseline were observed in both STEMI and NSTEMI and also in patients with a prescription statin versus no prescription stain at discharge. The results were consistent across study subgroups: age, gender, race, normal versus low LVEF, revascularisation versus no revascularisation with PCI. The findings of narrow 95% CIs represent a strong prognostic significance of baseline HLP for 5-year survival. The results of this study also reaffirm the benefits of statins in secondary prevention after AMI.^27^ A third of patients with AMI were discharged with no statin prescription and only 1 in 10 received high-intensity statin therapy attributable to multiple factors. In 2002, the National Cholesterol Education Programme Adult Treatment Panel III guideline recommended treating to specific LDL-C targets.^13^ However, in 2013, the American College of Cardiology (ACC) and the American Heart Association (AHA) expanded statin treatment to all adults with AMI regardless of LDL-C targets.^23^ Subsequently, rates of overall and high-intensity stain prescription in rates substantially increased in the post ACC/AHA guideline period in particular.^28 29^ In an analysis of filled statin prescription of a large US population (>1 million) in post ACC/AHA guideline period, only 62% had their prescription filled after a recent acute coronary syndrome.^30^ Physician and patient preferences and then practice patterns might be accountable for lower rates of statin prescription among patients with AMI between 2001 and 2011. Furthermore, in the USA, the prescription pattern of statin and statin by intensity varies with the geographical region,^31^ with current trends approaching those of continental Europe.^32^ We found that less than a third of study population had high adherence rate to statins (MPR ≥80%). Patients who were adherent to prescription statins 80% or more days during the 5-year of follow-up period were at lower risk of death than those with lower rates of adherence to stain. In further data analysis with other lipid fractions for 5-year mortality after index hospitalisation for AMI, only non-HDL-C, similar to LDL-C, was found to have an inverse association.

### Comparative studies in the clinical context

Patients hospitalised with CAD, showed wide variations in the ranges in admission LDL-C level with 50% having LDL- level <100 mg and 17.6% having a level <70 mg/dL.^33^ Evidence is lacking on direct association between the ranges in LDL-C levels and mortality risk. A number of early epidemiological studies demonstrated that HLP or elevated LDL-C levels were associated with increased cardiovascular events and mortality.^34 35^ This relationship was subsequently supported by several lipid-lowering clinical trials that demonstrated an association between LDL-C lowering and cardiovascular risk reduction.^36^ In contrast, several recent clinical trials and reports from large registry databases suggest that HLP is rather protective once a patient has developed AMI. Three large registry-based studies with the combined population of 211 309 patients found that patients with HLP or those in the high LDL-C quartiles had lower mortality compared with those with no HLP or in the lowest LDL-C quartile.^6 7 9^ In a similar analysis of 2465 patients, higher remnant lipoprotein cholesterol levels were associated with lower 2-year mortality after AMI.^5^ A prospective cohort study demonstrated that a lower admission LDL-C (<105 mg/dL) was associated with a reduced 3 years survival compared with those with elevated LDL-C.^37^ The findings of these studies were consistent with our results.

Lipid-lowering trials demonstrated that the relationship between follow-up cholesterol concentration during therapy and outcome was non-linear and independent of baseline cholesterol concentration.^38–40^ Although angiographic studies showed slowing of the progression of atherosclerotic lesion with diet and drug therapy, evidence is lacking for quantitative improvement in coronary artery lesions in relation to degree of lowering of LDL concentration achieved during therapy.^39 41^ Professional guidelines also emphasise the importance of adherence to stain therapy, which is low and declines with time.^11^ Our results compliment previously published studies that higher level of adherence to statins improves postmyocardial infarction outcomes.^42^

### Potential mechanisms of survival benefits with HLP

Underlying mechanisms for survival benefit conferred by HLP among patients with AMI as observed in the present study are not fully understood. Cholesterol level and its association with mortality may vary according to age and concomitant CCs. For instance, cholesterol level decreases with increasing age and the strength of its association with mortality either decreases or shows even inverse association with advancing age.^3 43 44^ The effect of HLP may be attenuated in the presence of other strong competing risk factors.^45^ While most studies focused on composite endpoints, a limited number of studies specifically examined the effect of LDL-C lowering by statin on all-cause mortality in patients with post-AMI. In 2014, National Institute for Health and Care Excellence of the United Kingdom sponsored an evidence review for the effect of LDL-C lowering by statin on all-cause mortality among patients with AMI. This systematic review (15 studies, n=60 106) found that the effect size was too small to be of clinical importance (https://www.ncbi.nlm.nih.gov/books/NBK248067/pdf/Bookshelf_NBK248067.pdf).

Current recommendations for statin therapy in AMI are mainly based on clinical trials of statins in persons who have had limited number of CCs. Additionally, the secondary prevention trials of statins were mainly conducted predominantly in middle-aged men.^46^ It is unclear whether these results are generalisable for all-cause mortality and across older patients with other life-limiting conditions. An increase in proportion of deaths from non-cardiovascular conditions with differential association with baseline HLP may account for an inverse association of HLP with all-cause mortality.^47^ The lowering of LDL-C with statin had no clear benefit in patients with AMI with at least certain comorbidities.^48 49^

Recent studies found striking differences in associations between *de novo* versus statin-mediated low LDL-C levels and postinfarction mortality.^50^ Furthermore, not all LDL-C lowering strategies are comparable in reducing the clinical outcomes in AMI. Emerging evidence suggests that LDL-C reduction by mechanisms other than enhanced clearance by LDL receptors was not associated with mortality reduction.^51 52^ HLP may lead to AMI earlier in the atherosclerotic disease process resulting in seemingly greater longevity in post-AMI follow-up, thereby introducing lead time bias.

### Strengths and limitations

This study has several strengths. First, high level of case ascertainment for incident events and prompt mortality update allowed precise estimation of mortality risks. Second, propensity score matching to balance observed patient characteristics enabled further control of potential differences. The study also has a number of important limitations. These included, several unmeasured confounders, reliance on ICD-9-CM codes to identify study cohort and CCS codes to assess coexisting CCs, and lack of data on subsequent acquisition of these conditions during the follow-up. The pre-existing HLP and CCs were physician diagnosed during index hospitalisation rather than being assigned by study investigators. The data were limited to patients hospitalised for AMI from 2001 through 2011 with suboptimal rates of prescription statin on discharge. The 2013 ACC/AHA guidelines may have influenced the prescription and adherence rates of statin among contemporary patients with AMI. Despite these limitations, the findings of the present study can be extended to hospital-based AMI population at large.

## Conclusions

Our findings in propensity score matched cohorts suggest that concomitant HLP, compared with no HLP, was associated with a decrease in all-cause mortality, potentially prolongs survival and adds life years over 5-year follow-up after AMI. These findings were independent of statin therapy and remained consistent across STEMI and NSTEMI subgroups and among patients with no prescription statin at discharge. Our results provide further support for the use of statin regardless of baseline LDL-C to reduce all-cause mortality to prolong overall survival after incident AMI. Our findings also underscore the importance of close adherence to statin therapy to improve postmyocardial infarction survival. We recommend future studies to clearly understand the effect of HLP and statin versus non-stain-induced LDL-C levels on all-cause mortality following incident AMI.
