# Supplementary material for: Association of hyperlipidaemia with 5-year survival after hospitalisation for acute myocardial infarction: a propensity score matched analysis
Source: Open Heart. 2020 Mar 17;7(1):e001163. doi: 10.1136/openhrt-2019-001163 (PMC7078940; doi:10.1136/openhrt-2019-001163)
Supplement: Supplementary data [file openhrt-2019-001163supp001.pdf]

Supplementary Material

Title:

Association of hyperlipidemia with 5-year survival after hospitalization for acute myocardial infarction: A propensity-score matched analysis

Study coauthors:
Mohammed Yousufuddin, Ye Zhu, Ruaa Al Ward, Jessica Peters, Taylor Doyle, Kelsey Jensen, Zhen Wang, Mohammad Hassan Murad

Supplement Table 1.. Page 2
Supplement Table 2.. Page 3
Supplement Table 3.. Page 5
Supplement Figure 1... Page 6
Supplement Figure 2... Page 7

Yousufuddin M, *et al. Open Heart* 2020; 7:e001163. doi: 10.1136/openhrt-2019-001163

Supplement Table 1. *International Classification of Diseases, Ninth Revision, Clinical Modification* codes for three index conditions used in the study

Diagnosis	*ICD-9-CM* Codes
Acute myocardial infarction	410.00, 410.01, 410.10, 410.11, 410.20, 410.21, 410.30, 410.31, 410.40, 410.41, 410.50, 410.51, 410.60, 410.61, 410.70, 410.71, 410.80, 410.81, 410.90, 410.91
Hypercholesterolemia	272.0, 272.1, 272.2, 272.3, 272.4, 272.5

Yousufuddin M, *et al. Open Heart* 2020; 7:e001163. doi: 10.1136/openhrt-2019-001163

Table 2: Baseline patient characteristics not incorporate

		All Patients n = 7,071			Propensity score-matched patients n = 4,182		
		Hyperlipidemia N = 4809	No hyperlipidemia N = 2262	P value	AMI with hyperlipidemia N = 2091	AMI with no hyperlipidemia N = 2091	P value
**Clinical presentation**	Chest pain, n = (%)	4,379 (91)	1,760 (78)	<0.0001	1,853 (89)	1647 (79)	<0.0001
	Dyspnea, n = (%)	2,587 (54)	1,133 (50)	<0.0001	1,010 (48)	1061 (51)	0.1147
	Weakness, n = (%)	2,669 (55)	883 (39)	<0.0001	845 (40)	837 (40)	0.8008
	Syncope, n = (%)	309 (6)	187 (8)	<0.0001	122 (6)	173 (8)	0.0021
**Comorbid conditions**	Depression, n = (%)	305 (6)	75 (3)	<0.0001	65 (3)	75 (4)	0.3868
	Arthritis, n = (%)	204 (4)	62 (3)	0.0017	80 (4)	61 (3)	0.1048
	Arrhythmia, n = (%)	1080 (23)	638 (28)	<0.0001	457 (22)	583 (28)	<0.0001
	Asthma, n = (%)	173 (4)	86 (4)	0.7086	46 (2)	40 (2)	0.5162
	Osteoporosis, n = (%)	106 (2)	36 (2)	0.0848	43 (2)	35 (2)	0.3628
	Dementia, n = (%)	97 (2)	58 (3)	0.1545	42 (2)	55 (3)	0.1801
	Schizophrenia, n = (%)	20 (0.5)	14 (0.1)	0.2790	8 (0.4)	13 (0.6)	0.2731
	Hepatitis, n = (%)	17 (0.5)	17 (0.7)	0.0254	7 (0.3)	16 (0.8)	0.0595
	HIV, n = (%)	4 (0.07)	1 (0.04)	0.5610	2 (0.1)	1 (0.05)	0.5641
	Autism spectrum, n = (%)	0	0		0	0	
	Substance use, n = (%)	173 (3.6)	89 (3.8)	0.7078	54 (3)	85 (4)	0.0074
**LVEF**	LVEF % mean ± SD	48 ±15	52 ±13	<0.0001	50 (14)	48 (15)	0.0091
	LVEF ≤ 50% n =			<0.0001	815 (49)	839 (50)	0.3767
	LVEF, missing n = (%)	854 (18)	471 (21)		426 (20)	429 (20)	0.3767
**Lipid fractions mg/dl**	TC	188 (50)	155 (42)	<0.0001	194 (46)	156 (42)	<0.0001
	TC, ≥201	2,821 (64)	1,587 (89)	<0.0001	823 (42)	201 (12)	<0.0001
	TC, missing, n = (%)	398 (8)	472 (21)		129 (6)	430 (21)	
	HDL-C	44 ± 13	44 ± 15	0.9098	45 ± 13	44 ± 15	0.0044
	HDL-C ≤ 45, n = (%)	2,828 (62)	1,132 (62)	0.8745	845 (38)	652 (42)	0.0138
	HDL-C, missing n = (%)	233 (5)	424 (18)		84 (4)	381 (18)	
	Non-HDL-C	144 ± 49	111 ± 41	<0.0001	148 ± 45	111 ± 41	<0.0001
	Non-HDL-C ≥130, n = (%)	2,716 (62)	402 (23)	<0.0001	1,310 (67)	379 (23)	<0.0001
	Non-HDL-C, missing n = (%)	406 (8)	489 (22)		133 (6)	445 (21)	
	Triglyceride	151 ± 133	144 ± 139	0.0380	145 ± 95	145 ± 141	0.9970

Yousufuddin M, *et al. Open Heart* 2020; 7:e001163. doi: 10.1136/openhrt-2019-001163

	Triglyceride ≥176, n = (%)	1,186 (27)	394 (22)	<0.0001	479 (24)	361 (22)	0.0563
	Triglyceride, missing n = (%)	402 (8)	473 (21)		133 (6)	433 (21)	
**Revascularization**	PCI, n = (%)	2,733 (57)	1,010 (45)	<0.0001	1,146 (55)	954 (46)	<0.0001
	CABG, n = (%)	769 (16)	347 (15)	0.663	355 (17)	312 (15)	0.0694
**Circulatory support**	LVAD	53 (1)	47 (2)	<0.0001	12 (0.6)	19 (0.9)	0.2050
**Drug treatment**	Statin, n = (%)	2714 (56)	795 (35)	<0.0001	1266 (55)	1044 (45)	<0.0001
	High-intensity statin, n = (%)	192 (8)	14 (1.9)	<0.0001	161 (11)	128 (9)	0.0413
	Ezetimibe, n = (%)	49 (1)	5 (0.2)	<0.0001	11 (0.5)	5 (0.2)	-
	Gemfibrozil, n = (%)	102 (2)	26 (1.2)	<0.0001	33 (1.6)	23 (1)	-
	Niacin, n = (%)	138 (2.9)	23 (1)	<0.0001	37 (1.8)	23 (1)	-

Abbreviations: AMI, acute myocardial infarction; BMI, body max index; CABG, coronary artery bypass surgery; CKD, chronic kidney disease; COPD, chronic obstructive pulmonary disease; HDL-C, high-density lipoprotein cholesterol; HIV, human immunodeficiency virus infection; LDL, low- density lipoproteins; LOS, length of stay; LVAD, left ventricular assist device; LVEF, left ventricular ejection fraction; PCI, percutaneous coronary intervention; SD, standard deviation; TC, total cholesterol.

Yousufuddin M, *et al. Open Heart* 2020; 7:e001163. doi: 10.1136/openhrt-2019-001163

Supplement Table 3. Association of hyperlipidemia and no hyperlipidemia with 5-year mortality across subgroups according to age, gender, race, left ventricular ejection fraction, statin therapy, and revascularisation strategy

Characteristics	Subgroups	HR (95% CI)	P value
Age	<65 year	0.60 (0.49 – 0.73)	<0.0001
	≥ 65 year	0.72 (0.66 – 0.79)	<0.0001
Gender	Male	0.68 (0.61 – 0.75)	<0.0001
	Female	0.76 (0.67 – 0.87)	<0.0001
Race	White	0.70 (0.64 – 0.77)	<0.0001
	Non-white	0.75 (0.57 – 0.98)	0.0356
Left ventricular ejection fraction	≥ 50%	0.69 (0.60 – 0.79)	<0.0001
	≤ 49%	0.69 (0.61 – 0.78)	<0.0001
Statin therapy	Statin	0.66 (0.59 – 0.74)	<0.0001
	No Statin	0.81 (0.72 – 0.92)	0.0012
Revascularization	No PCI	0.72 (0.65 – 0.81)	<0.0001
	PCI	0.73 (0.64 – 0.82)	<0.0001
	No CABG	0.69 (0.63 – 0.76)	<0.0001
	CABG	0.84 (0.68 – 1.04)	0.1113

Abbreviations: CABG, coronary artery bypass surgery; CI, confidence interval; HR, hazard ratio; PCI, percutaneous coronary intervention.

Yousufuddin M, *et al. Open Heart* 2020; 7:e001163. doi: 10.1136/openhrt-2019-001163

Supplement Figure 1. STROBE (Strengthening the Reporting of Observational Studies in Epidemiology) flow diagram of the process of selection of study cohorts

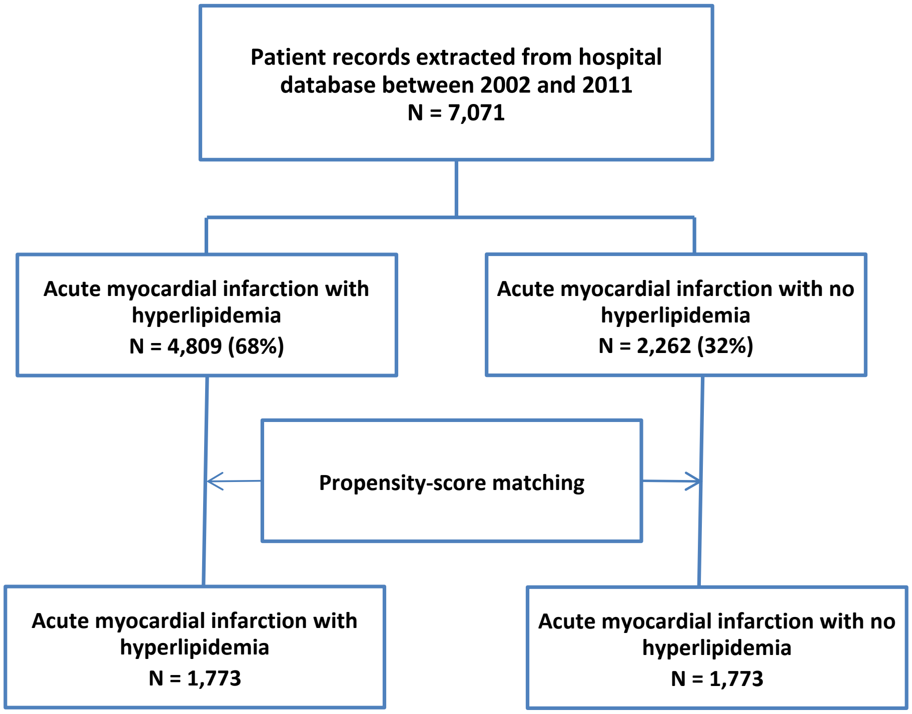

Supplement Figure 2. Survival to discharge after acute myocardial infarction. Kaplan-Meier Survival estimates of patients with concurrent hyperlipidemia vs no hyperlipidemia

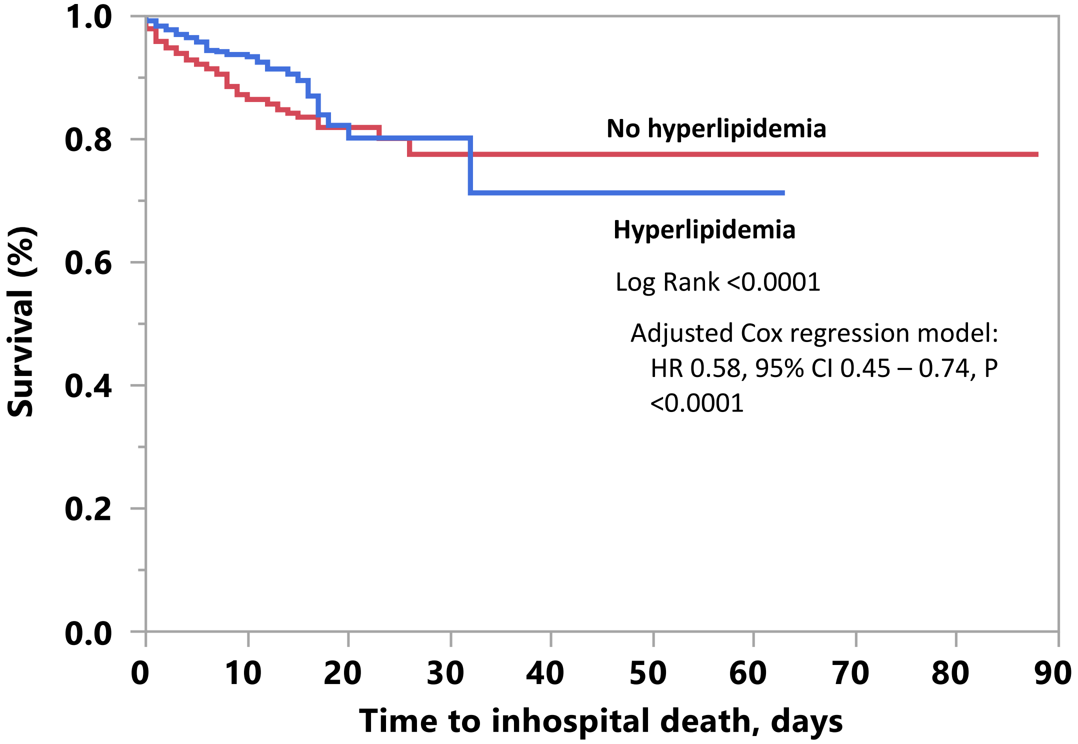

Yousufuddin M, *et al. Open Heart* 2020; 7:e001163. doi: 10.1136/openhrt-2019-001163
